# The development of a TED-Ed online resident research training program

**DOI:** 10.3402/meo.v19.26128

**Published:** 2014-12-18

**Authors:** Katherine A. Moreau, Catherine M. Pound, Beth Peddle, Jaclyn Tokarewicz, Kaylee Eady

**Affiliations:** 1 Children's Hospital of Eastern Ontario Research Institute, Ottawa, Ontario, Canada; 2 Department of Pediatrics, Faculty of Medicine, University of Ottawa, Ontario, Canada; 3 Faculty of Education, University of Ottawa, Ottawa, Ontario, Canada; 4 Division of Pediatrics, Children's Hospital of Eastern Ontario, Ottawa, Ontario, Canada; 5 Telfer School of Management, University of Ottawa, Ottawa Ontario, Canada; 6 School of Rehabilitation Sciences, Faculty of Health Sciences, University of Ottawa, Ottawa, Ontario, Canada

**Keywords:** residents, scholar, e-learning, pediatrics

## Abstract

**Background:**

Pediatric health research is important for improving the health and well-being of children and their families. To foster the development of physicians’ research competencies, it is vital to integrate practical and context-specific research training into residency programs.

**Purpose:**

To describe the development of a resident research training program at one tertiary care pediatric academic health sciences center in Ontario, Canada.

**Methods:**

We surveyed residents and pediatricians/research staff to establish the need and content for a resident research training program.

**Results:**

Residents and resident research supervisors agreed or strongly agreed that research training is important for residents. However, few residents and supervisors believed that their academic health sciences center provided adequate training and resources to support resident research. As such, an online resident research training program was established. Residents and supervisors agreed that the program should focus on the following topics: 1) critically evaluating research literature, 2) writing a research proposal, 3) submitting an application for research funding, and 4) writing a manuscript.

**Discussion:**

This highly accessible, context-specific, and inexpensive online program model may be of interest and benefit to other residency programs as a means to enhance residents’ scholarly roles. A formal evaluation of the research training program is now underway.

Pediatric health research is vital for improving the health and well-being of children and their families. By facilitating pediatric residents’ research abilities, clinical advancements will continue in pediatric medicine, more graduates will pursue physician-scientist careers, and future pediatricians will have the research skills needed to supervise the research initiatives of medical trainees (1–3). To foster the development of competencies within research, professional associations and academic centers that oversee medical education have acknowledged the importance of scholarly activities as a necessary component of residency training. The Royal College of Physicians and Surgeons of Canada identified the scholar role – the ability to create, disseminate, apply, and translate medical knowledge – as one of seven key competencies that residents must attain through their postgraduate training (4). The Accreditation Council for Graduate Medical Education also supports research as an important component of residency training programs by requiring residents to participate in scholarly activities and demonstrate their understanding of the basic principles of research, including how research is conducted, evaluated, explained to patients, and applied to patient care (5).

However, despite consensus that research competency is highly valuable, pediatric academic leaders have articulated concern that pediatric residency programs are failing to expose residents to research training, and thus, neglecting to foster the development of both pediatric researchers and pediatricians who can evaluate and apply scientific findings to clinical practice (1, 6–8). Research also shows that many residency programs are struggling to incorporate research training because they lack practical, timely, and relevant research training resources (9). To help mitigate these challenges and enhance residents’ access to practical and context-specific research training and resources, we initiated the development of a resident research training program. In this article, we describe the need for and development of this program at one tertiary care pediatric academic health sciences center in Ontario, Canada.

## Methods

### Needs assessment

To establish the need for and develop the resident research training program, we surveyed residents (*N*=38) and pediatricians/research staff eligible to supervise residents’ research projects (*N*=105) at a pediatric academic health sciences center in Ontario, Canada. We developed two online needs assessment surveys (delivered via Fluid^©^ surveys): one for the residents and another for the resident research supervisors. Using previous studies that examined the research training needs of residents in other specialties (1, 3), we developed the items for these two similar surveys. The 13 closed-ended item resident survey investigated residents’ demographic characteristics, their perceptions of research, research topics that would be most beneficial to their required research projects, and their preferences for online learning strategies. Likewise, the six closed-ended item resident research supervisor survey explored these individuals’ demographic characteristics, their perceptions of research, and research topics that would be most beneficial to the research projects that are required of residents. All data were analyzed using frequencies and percentages in SPSS 22 (10). Using the findings from the needs assessment surveys, we then constructed the resident research training program.

## Results

### Needs assessment

The residents’ needs assessment survey was completed by 28 residents (74%), with the majority (80%) being female. Of those that responded, approximately 32% were in first-year residency, 32% were in second year, 21% were in third year, and 14% were in fourth year. Most respondents (79%) had some research training prior to residency. When asked to indicate the various places where they received this training, 68% stated their undergraduate degree, 73% indicated medical school, and 14% selected Master's degree. At the time of the survey, the majority of residents (71%) were working on their resident research projects, whereas the remainder had completed their projects. Half of the respondents (50%) were looking to obtain an academic appointment upon completion of their residency training. The resident research supervisor survey was completed by 58 individuals (55%). Of those, 60% were assistant professors, 33% were associate professors, and 7% were full professors. Almost one-fourth (21%) identified themselves primarily as clinician-scientists, and the rest (79%) as physicians. Slightly more than 80% of both residents and resident research supervisors agreed or strongly agreed that research training was important for residents. However, only one-fourth (26%) of supervisors and one-third (32%) of residents believed that their tertiary care pediatric academic health sciences center was supportive of resident research (i.e., provided sufficient resources and training for it).

When asked to select the top four research topics that would be most beneficial to residents’ research projects, residents and supervisors were in consensus, as they both selected the following topics: 1) critically evaluating research literature, 2) writing a research proposal, 3) submitting an application for research funding, and 4) writing a manuscript. When residents were asked if they would use an online resource dedicated to their research training, 61% said they would, 29% said they would not, and 11% said they were unsure. Of those who said they would use an online resource, 88% thought that an online educational session should last 10–40 min.

### Online resident research training program

Because the majority of the residents stated that they would use an online resource dedicated to their research training, we opted for an online program. Based on the aforementioned needs assessment results, we created four foundational educational YouTube videos on the following topics: 1) critically evaluating research literature; 2) writing a research proposal; 3) submitting an application for research funding; and 4) writing a manuscript. Each educational video includes a 10-min, narrated PowerPoint presentation (created by a topic expert from our center's research institute) and a 5-min video interview with another topic-matter expert from our center. The videos are designed to introduce residents to important research terms and concepts, explain organizational research policies and procedures, and introduce them to local topic-matter experts who can assist them in the development of their research projects.

To enhance the educational experience for residents and disseminate and evaluate this training program, we are using TED-Ed – a new free platform for sharing educational lessons and tracking their impact on learners (11). It is an extension of TED, which is a non-profit organization devoted to spreading ideas, usually in the form of short, powerful talks (12). TED-Ed allows us to take our educational YouTube videos and create customized lessons around them. These lessons include discussion forums, reaction questions, knowledge testing questions based on the video content, and supplemental materials (e.g., journal articles, books, and websites) about the specific topics. Using TED-Ed, we are now distributing the lessons, via a closed group (for the purposes of program evaluation), to our participating residents, assessing their discussion postings, their reactions to the lessons, their responses to the knowledge testing questions, and tracking the number of times they access the various lessons. To ensure timely access, these lessons are available to the residents via their smartphones and other portable electronic devices. Each lesson is non-cumulative, so that skipping one lesson will not make future lessons incomprehensible, as a way of accommodating residents’ busy schedules. This also means that residents can view only those lessons that are of specific interest and relevance to them and their research projects.

## Discussion

Although similar online research training programs exist for health professionals, our resident research training program is created to specifically target the research training needs of residents affiliated with our tertiary care pediatric academic health sciences center. One of the major strengths of this program, which may be especially appealing to other residency and undergraduate medical education programs, is the e-learning component, as it provides learners with increased accessibility to research training. To date, the TED-Ed platform has also been advantageous because it allows us to embed selected program evaluation activities into the program itself and customize the lessons to the needs of our residents. Moreover, because TED-Ed is free and we relied heavily on in-kind contributions of local experts, we were able to create this online program on a modest budget and minimal resources. To develop our program, we only required one student research assistant for approximately 45 hours to: 1) administer the two online needs assessment surveys and summarize the findings, 2) format the narrated PowerPoint presentations, 3) film the 5-min video interviews with topic-matter experts from our center, and 4) upload the content to YouTube and TED-Ed. As such, we believe that this highly accessible, context-specific, and inexpensive online program model may be of interest and benefit to other residency programs. After conducting their own needs assessment, which may be informed by our needs assessment survey items, residency programs from other specialties can use our video and lesson templates to create their own online resources that meet their residents’ specific research training needs as well as the scholarly requirements of their academic centers. Moreover, undergraduate medical education programs could follow a similar process to create specific online research training resources tailored to their medical students as well as their curricula expectations for research. Such efforts will enhance research capacity and learners’ fulfillment of scholarly roles.

To further develop resident research capacity at our center, we are developing a variety of face-to-face activities (e.g., research workshops, interactive academic half day research sessions, research in-progress rounds, and resident research mentorship groups). These activities will complement our online program, allow residents to gain practical hands-on research experience, and address the needs of the 29% of respondents who said they would not use an online resource dedicated to their research training. Moreover, we are beginning to systematically evaluate our online resident research training program. To do so, we are exploring residents’ reactions to the program and the extent to which they are accessing it. We are also examining what they have learned from the program and determining what additional research training is still needed. This evaluation will help us improve the program, develop additional TED-Ed lessons, and identify potential topics for the complementary face-to-face activities. To facilitate these evaluation efforts we are using residents’ responses to the above-mentioned discussion forums, reaction questions, and knowledge testing questions that are embedded into our TED-Ed lessons.
